# Protective Mechanisms of EGCG in Mitigating Oxidative Stress and Liver Toxicity From Cigarette Smoke‐Induced Damage

**DOI:** 10.1002/fsn3.70472

**Published:** 2025-07-07

**Authors:** Kağan Ağan, Şerif Demir, Recep Özmerdivenli, Aydan Fülden Ağan, Ali Tuğrul Akın, Merve Alpay, Özge Beyazçiçek, Ersin Beyazçiçek, Mehmet Ali Sungur

**Affiliations:** ^1^ Experimental Animals Application and Research Center Düzce University Düzce Türkiye; ^2^ Faculty of Medicine, Department of Physiology Düzce University Düzce Türkiye; ^3^ Faculty of Medicine, Department of Physiology Aydın Adnan Menderes University Aydın Türkiye; ^4^ Faculty of Medicine, Department of Medical Biology İstinye University İstanbul Türkiye; ^5^ Faculty of Medicine, Department of Medical Biochesmitry Düzce University Düzce Türkiye; ^6^ Faculty of Medicine, Department of Biostatistics and Medical Informatics Düzce University Düzce Türkiye

**Keywords:** cigarette smoke, epigallocatechin gallate (EGCG), in vivo, liver toxicicity

## Abstract

Exposure to cigarette smoke leads to an increase in oxidative stress within the body, resulting in both an elevated oxidant burden and a compromised antioxidant defense system. This imbalance creates a significant risk factor for various diseases by promoting cellular damage, inflammation, and toxicity. The oxidants present in cigarette smoke are considered the primary contributors to these pathological conditions. Supporting the antioxidant system with specific bioactive compounds may help mitigate the toxic effects caused by cigarette smoke. In this study, the effects of EGCG pre‐administration on the antioxidant system were evaluated under both acute and chronic exposure conditions to cigarette smoke. Different doses of EGCG were administered to determine its potential role in oxidative stress regulation, and histopathological examinations and antioxidant enzyme levels were assessed. The findings demonstrated that while acute EGCG administration did not significantly improve antioxidant enzyme activity, chronic administration of EGCG at a dose of 50 mg/kg effectively increased antioxidant enzyme production, reduced oxidative stress, and liver injury. In the presence of cigarette smoke, EGCG contributed to the stabilization of oxidative stress markers. However, chronic EGCG administration in the absence of oxidative stressors requires further investigation to assess its impact on other organs. EGCG appears to be a promising candidate for alleviating the adverse effects of external oxidant exposure and mitigating oxidative stress. However, its long‐term application and potential side effects in different physiological conditions should be explored further examinations. Although acute EGCG application did not enhance antioxidant enzyme levels, it unexpectedly elevated oxidative stress, emphasizing the need for more comprehensive studies to clarify its mechanisms and optimize its usage. We further identify the principal underlying mechanisms involved in this process.

## Graphical abstract

The chronic administration of EGCG has been demonstrated to effectively mitigate the oxidative stress that is induced by chronic exposure to the harmful components in cigarette smoke, thus potentially providing a protective effect. It is essential to acknowledge that the beneficial effects of EGCG administration on the antioxidant defense system require a specific duration of time to manifest fully. The chronic application of EGCG has been shown to result in a significant increase in the levels of the antioxidant enzymes glutathione peroxidase (GPx) and superoxide dismutase (SOD) and chronically treated with EGCG, especially at the dose of 50 mg/kg, displayed relatively healthier liver tissue morphology.

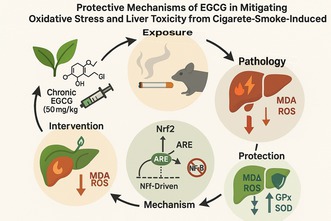

## 1 Introduction

Smoking represents a global public health issue, being a significant environmental risk factor associated with numerous diseases. It causes the death of 6 million people every year (U.S. Department of Health and Human Services 2012). It creates a severe socio‐economic burden on countries due to its direct and indirect effects (Hay et al. 2017; Vestbo et al. 2013).

Exposure to cigarette smoke also affects passive smokers, contributing to this economic burden as much as smoking and further exacerbating health‐related complications. Cigarette smoke contains more than 4000 different chemicals and reactive oxygen species (ROS) known to have carcinogenic effects (Church and Pryor 1985). These cause lipid peroxidation and the development of oxidative stress (Rahman et al. 2002). Oxidative stress induced by cigarette smoke causes pathogenesis in organs (Rueff‐Barroso et al. 2010). ROS are known to cause breast cancer (Lee et al. 2010). It also causes diseases in organs that are not in direct contact with cigarette smoke, such as the liver (IARC 2004) and colorectal (Botteri et al. 2008) cancer types. Smoking and exposure to cigarette smoke increase the amount of toxins entering the body and cause liver cell damage in humans (El‐Zayadi and Abdel‐Rahman El‐Zayadi 2006). It also causes respiratory (U.S. Department of Health and Human Services 2012) and cardiovasculer (Lee et al. 2010) system disorders.

The use of cigarettes is considered a significant source of oxidative stress as it increases the production of oxidant molecules in the body (Rahman et al. 2002). This condition plays a critical role in cellular damage and the development of various diseases. Besides the increase in ROS caused by external factors, ROS is formed as a result of metabolism in cells in the body. It affects many physiological events, e.g., cell proliferation, energy production, gene transcription, and expression. Cell damage occurs as a result of increased oxidative stress (Pervaiz et al. 2009; Mittal et al. 2014). When there is an increase in oxidants entering the body during inflammation, secretion from macrophages and netrophils occurs (Kirkham and Rahman 2006). In addition, levated oxidant levels contribute to cellular damage by causing oxidative modifications in critical biomolecules, such as proteins and DNA (Bandyopadhyaya et al. 2008). These molecular alterations can lead to intracellular accumulation and impairment of cellular functions, ultimately paving the way for lipid peroxidation as a key step in the oxidative damage process. Oxidation of unsaturated fatty acids is called lipid peroxidation (Churg and Cherukupalli 1993). As a result of this oxidation, reactive aldehydes such as 4‐hydroxynonenal (4‐HNE), malondialdehyde (MDA), propanal, and hexanal, which are indicators of toxicity, are formed (Ayala et al. 2014).

An increase in the amount of carboxyhaemoglobin causes the other toxic effect. The increase in carboxyhaemoglobin reduces the oxygen‐carrying capacity. This leads to an increase in red blood cells (RBCs). While the amount of iron absorbed for the production of RBCs increases (increased iron absorption), the amount of iron also increases due to increased RBC destruction (catabolic iron). Increased iron accumulates in the liver and causes increased oxidative stress in hepatocytes (Gutteridge and Halliwell 1989). It also causes liver toxicity by activating mechanisms such as apoptosis (Bandiera et al. 2020). Iron‐induced oxidative stress and hepatotoxicity can impair the liver's ability to perform its vital functions, including detoxification, metabolism, and biotransformation of various substances such as drugs, alcohol, and toxins.

Additionally, exposure to toxic agents like cigarette smoke further exacerbates liver dysfunction by triggering oncogenic and immunologically adverse effects (El‐Zayadi and Abdel‐Rahman El‐Zayadi 2006). Cigarette smoke disrupts cellular structure by affecting DNA expression (Xiao et al. 2015; Kopa and Pawliczak 2018; Zhao et al. 2009). Its constituents, such as arsenic, formaldehyde, and nitrosamines, induce DNA strand breaks and recruit DNA methyltransferases (DNMTs) to sites of damage, leading to altered gene expression profiles and compromised cellular architecture (Silva and Kamens 2020). Smoking increases the risk of developing hepatocellular carcinoma in patients with chronic liver disease (Hara et al. 2008). Oxidative stress has been shown to cause total DNA damage in liver tissue, as demonstrated in studies investigating genotoxic effects in rat liver (Bandyopadhyaya et al. 2008). Exposure to cigarette smoke disrupts the balance between the oxidant‐antioxidant system, leading to depletion of antioxidants and an increased oxidative stress within the body. Oxidative stress plays a critical role in the activation of NF‐κB signaling, leading to the production of inflammatory enzymes, cytokines, and chemokines (Kode et al. 2006), which in turn contribute to further ROS formation and cellular damage (Wan and Lenardo 2010). In this context, dietary polyphenols such as tea catechins, curcumin (Feng et al. 2019; Weisberg et al. 2008), resveratrol (Sgambato et al. 2001), and honokiol (Khalid et al. 2018) have been extensively studied for their antioxidant and anti‐inflammatory properties. Polyphenols exert their protective effects by scavenging free radicals and inhibiting NF‐κB signaling, thus reducing inflammation and oxidative stress (Rahman 2008).

Since the damage caused by cigarette smoke is due to the oxidant substances it contains, people with smoking‐related diseases are likely to be protected by substances such as polyphenols, which have a positive effect on the antioxidant system (Pryor and Stone 1993). Among dietary sources, tea polyphenols—particularly epigallocatechin gallate (EGCG)—have drawn attention for their potent antioxidant and anti‐inflammatory properties, showing potential to counteract cigarette smoke‐induced oxidative damage not only in the lungs but also in extrapulmonary organs such as the liver (Rudrapal et al. 2022). As tea has these polyphenols and widespread use (Food and Agriculture Organization of the United Nations 2018), it can be a potential molecule to prevent excessive oxidative stress (Rudrapal et al. 2022). The studies on its effects are being carried out increasingly (Food and Agriculture Organization of the United Nations 2018). Tea is a plant originating from China and Southeast Asia that has been cultivated and consumed for more than 2000 years. It is obtained from the leaves of the plant called *Camellia sinensis* (Ukers 1935) and is the 2nd most consumed beverage after water (Ouyang et al. 2020). Besides its widespread consumption, flavanols, including catechins, constitute 30% of the dry weight of fresh tea leaves. These leaves contain four important catechin groups: epicatechin (EC), epicatechin gallate (ECG), and the major tannins epigallocatechin (EGC) and epigallocatechin gallate (EGCG) (Rudrapal et al. 2024). Catechins are the colorless, water‐soluble, bitter substances responsible for astringency taste (Balentine et al. 1997). EGCG (48%–55%), EGC (9%–12%), ECG (9%–12%), and EC (5%–7%) are the most abundant catechins in green tea, respectively (Sano et al. 2001). While EC, ECG, EGC, and EGCG collectively constitute the major catechin groups in green tea, EGCG is notably distinguished from the other catechins by not only being a potent OH radical scavenger but also by significantly inhibiting NF‐κB activation—a critical pathway linking oxidative stress to chronic inflammation and carcinogenesis, thereby offering superior antioxidant and anti‐inflammatory properties (Rudrapal et al. 2024). This multifunctional capacity underscores EGCG's superiority over other catechins, which primarily exert antioxidant effects through free radical neutralization (Saito et al. 2007). Thanks to its dihydroxyl and trihydroxyl structures, EGCG is an effective free radical scavenger (Rice‐Evans 1999).

A study in 2020 demonstrated that EGCG administration following cigarette smoke exposure prevented the accumulation and formation of reactive oxygen species while also suppressing lipid peroxidation. By inhibiting NF‐κB activation, EGCG reduced the production of inflammatory mediators (Lakshmi et al. 2020). Furthermore, studies on aged rats have revealed that EGCG enhances the activity of antioxidant enzymes, including glutathione peroxidase (GPx), glutathione reductase (GR), superoxide dismutase (SOD), and catalase (CAT), while also increasing glutathione (GSH) levels and reducing malondialdehyde (MDA) levels (Srividhya et al. 2008). The mechanism underlying these effects involves the activation of the nuclear factor erythroid 2‐related factor 2 (Nrf2) transcription factor, which translocates to the cell nucleus and stimulates the antioxidant‐response element (ARE) enhancer region responsible for the gene expression of antioxidant enzymes (Chen et al. 2000; Wu et al. 2006; Itoh et al. 1999; Rubiolo et al. 2008).

This study aims to investigate the protective effects of EGCG pre‐administration by evaluating toxicity and antioxidant levels in acute and chronic exposure to cigarette smoke.

## 2 Material and Methods

### 2.1 Materials

Epigallocatechin gallate (EGCG) (Item No: 70935) was purchased from Cayman Chemical (Michigan, USA). Based on the company's recommendation, before the experiments started, the EGCG amounts required for each day's application were weighed, portioned, labeled, and stored at −20°C. Each EGCG quantity was completely dissolved in distilled water and homogenized before daily application. Aspartate aminotransferase (AST), alanine aminotransferase (ALT), Catalase (CAT), glutathione peroxidase (GPx), superoxide dismutase (SOD) and malondialdehyde (MDA) were purchased from Sunredbio, Shanghai Sunred Biological Technology (Shanghai, China). Hematoxylin and Eosin were purchased from Sigma Aldrich (St. Louis, MO).

### 2.2 Animals

All steps of our research, including the breeding stage, animal care, and the end of the experiment, were carried out at Düzce University Experimental Animals Application and Research Center (DÜDAM). The ethical permission required for our study was obtained from the Experimental Animals Local Ethics Committee of Düzce University, dated 16.04.2019, numbered 3/1.

In our study, 84 CD1 wildtype male mice of 2–3 months old and weighing 30 ± 5 g were used. Our experimental animals were cared for in an environment with a 12:12 automatic photoperiod, at 23°C room temperature, and 60% ± 5% humidity, with standard pellet feed and tap water available ad libitum throughout all life stages. Mice were given distilled water (EGCG Solvent) or EGCG intraperitoneally (i.p.) once daily. Administration of EGCG was at doses of 25 or 50 mg/kg. We formed acute and chronic experimental groups. Mice were divided into 12 groups (*n* = 7) as in Tables 1 and 2. Experimental groups were coded as follows: the first (lowercase) letter denotes exposure type (‘a’ for acute, ‘c’ for chronic), the subsequent uppercase letters indicate the exposure condition (‘V’ for vehicle/air, ‘CS’ for cigarette smoke), and the final number specifies the EGCG dose (0, 25, or 50 mg/kg per administration). At the end of acute or chronic experiments, mice were sacrificed for the collection of blood and liver samples.

**TABLE 1:** Design of the acute group application.

Groups' number	Groups' name	Application substance	Application method	Quantity of applications substance per animal	Period (minute/day) or Time (time/day) of applications substance per animal	Animal number
1	aV0	Ambient air	Inhalation	—	180 min/day	7
2	aCS0	Cigarette smoke	Inhalation	10 burned cigarettes' smoke	180 min/day	7
3	aV25	EGCG	IP	25 mg/kg	1time/day	7
		Ambient air	Inhalation	—	180 min/day	
4	aCS25	EGCG	IP	25 mg/kg	1time/day	7
		Cigarette smoke	Inhalation	10 burned cigarettes' smoke	180 min/day	
5	aV50	EGCG	IP	50 mg/kg	1time/day	7
		Ambient air	Inhalation	—	180 min/day	
6	aCS50	EGCG	IP	50 mg/kg	1time/day	7
		Cigarette smoke	Inhalation	10 burned cigarettes' smoke	180 min/day	

**TABLE 2:** Design of the chronic group application.

Groups' number	Groups' name	Application substance	Application method	Quantity of applications substance per animal	Period (minute/time/day) or Time (time/day) of applications substance per animal	Animal number
7	cV0	Ambient air	Inhalation	—	60 min/3time/21 day	7
8	cCS0	Cigarette smoke	Inhalation	3 burned cigarettes' smoke	60 min/3time/21 day	7
9	cV25	EGCG	IP	25 mg/kg	1time/21 day	7
		Ambient air	Inhalation	—	60 min/3time/21 day	
10	cCS25	EGCG	IP	25 mg/kg	1time/21 day	7
		Cigarette smoke	Inhalation	3 burned cigarettes' smoke	60 min/3time/21 day	
11	cV50	EGCG	IP	50 mg/kg	1time/21 day	7
		Ambient air	Inhalation	—	60 min/3time/21 day	
12	cCS50	EGCG	IP	50 mg/kg	1time/21 day	7
		Cigarette smoke	Inhalation	3 burned cigarettes' smoke	60 min/3time/21 day	

### 2.3 Cigarette Smoke Exposure Protocol

In this study, cigarette smoke exposure protocols were designed based on previous literature (Churg and Cherukupalli 1993; Drummond et al. 2016). In brief, commercially available cigarettes containing 0.7 mg nicotine, 7 mg carbon monoxide, and 7 mg tar per cigarette were lit in the smaller “smoke generation” chamber (25 × 15 × 33 cm). The resulting mainstream and sidestream smoke was continuously pumped into the larger animal chamber (60 × 40 × 40 cm) to ensure uniform exposure. Mice in the acute groups were exposed to smoke from 10 cigarettes burned consecutively over a single 180‐minute session, while chronic groups underwent three daily sessions of three cigarettes each (total nine cigarettes per day) for 21 consecutive days (Drummond et al. 2016). The duration of time allocated by these groups in the smoke room was calculated to ensure housing animals that were subjected to ambient air. Thus, it was ensured that all groups spent an equal amount of time in the smoke room. Control animals were housed in identical chambers supplied with filtered ambient air for the durations to maintain equivalent handling and environmental conditions.

### 2.4 Histopathological Analysis

Liver tissues were fixed in a 4% formaldehyde solution to preserve their structural integrity for histological examination. Following fixation, samples underwent a series of dehydration, clearing, and paraffin‐embedding steps. Thin tissue sections (5 μm) were obtained from paraffin blocks and subsequently stained for microscopic evaluation. Histopathological changes were assessed using a light microscope (Olympus BX51, Center Valley, PA), and representative photomicrographs were captured for documentation.

#### 2.4.1 Hematoxylin and Eosin (H&E) Staining

To prepare liver sections for staining, paraffin‐embedded samples were first incubated at 58°C for two hours to facilitate deparaffinization. After this step, sections were gradually rehydrated through a graded alcohol series and subsequently rinsed with tap water. Hematoxylin and eosin staining was then performed at room temperature to visualize tissue morphology. The stained sections were analyzed under a light microscope to evaluate histological alterations in liver tissues. Following the staining procedure, dehydration, clearing, and coverslipping steps were completed before microscopic evaluation.

#### 2.4.2 Liver Injury Scoring

Histopathological alterations in liver tissue were assessed based on specific criteria, including mononuclear cell infiltration, hemorrhage, irregular sinusoidal chords, and eosinophilic necrotic hepatocytes. A semi‐quantitative scoring system was applied as follows: 0 = no detectable pathology, 1 = 0%–25% involvement, 2 = 26%–45%, 3 = 46%–75%, and 4 = 76%–100% tissue involvement. The histological scores obtained from all experimental groups were analyzed quantitatively and subjected to statistical comparison to determine significant differences among groups.

### 2.5 Biochemical Analysis

Before surgery, a 200 mg/kg dose of ketamine and a 10 mg/kg dose of xylazine anesthetics were administered intraperitoneally to all mice. After checking paw reflexes, we ensured that all animals were under anesthesia. Then an incision was made from the median abdomen to the chin, and the sternum was removed. Blood was drawn using the exsanguination method, and animals were sacrificed. The collected blood was centrifuged at + 4°C and 4000 rpm for 10 min with the Nüve NF1200 device and stored at −86°C in an ultra‐low temperature deep freezer (Nüve DF590) until it was analyzed.

In our study, blood serums of animals were utilized for the ELISA assay. AST and ALT kits were used to assess liver toxicity, and CAT, GPx, and SOD kits were used for the antioxidant system, and MDA was used for oxidative stress. After applying the ELISA process steps, the Biotek 800 TS Absorbance Reader machine was used to read the plates. By using the absorbance and concentration values of the standard samples given with each individual kit, the equations of the curve that provide the condition closest to *R* ^2^ = 1 are obtained. For each sample, absorption values read at 540 nm in the ELISA were written into these equations, and concentrations were calculated.

### 2.6 Statistical Analysis

Statistical analysis of the data was performed using SPSS v.22 and the Statistica package programs. A three‐way factorial analysis of variance was used to evaluate the combined effect (interaction) of the mode of administration, administration, and dose. If the interaction was found significant, the LSD test was used for multiple comparisons. Descriptive statistics are summarized as a table in terms of mean and standard deviation. A statistical significance level of 0.05 was taken into account.

## 3 Results

To investigate the potential protective effect of acute or chronic EGCG administration on oxidative stress induced by cigarette smoke, we evaluated parameters related to oxidative stress in the present study.

### 3.1 EGCG Mitigates Histopathological Damage in Liver Tissue

**FIGURE 1:**
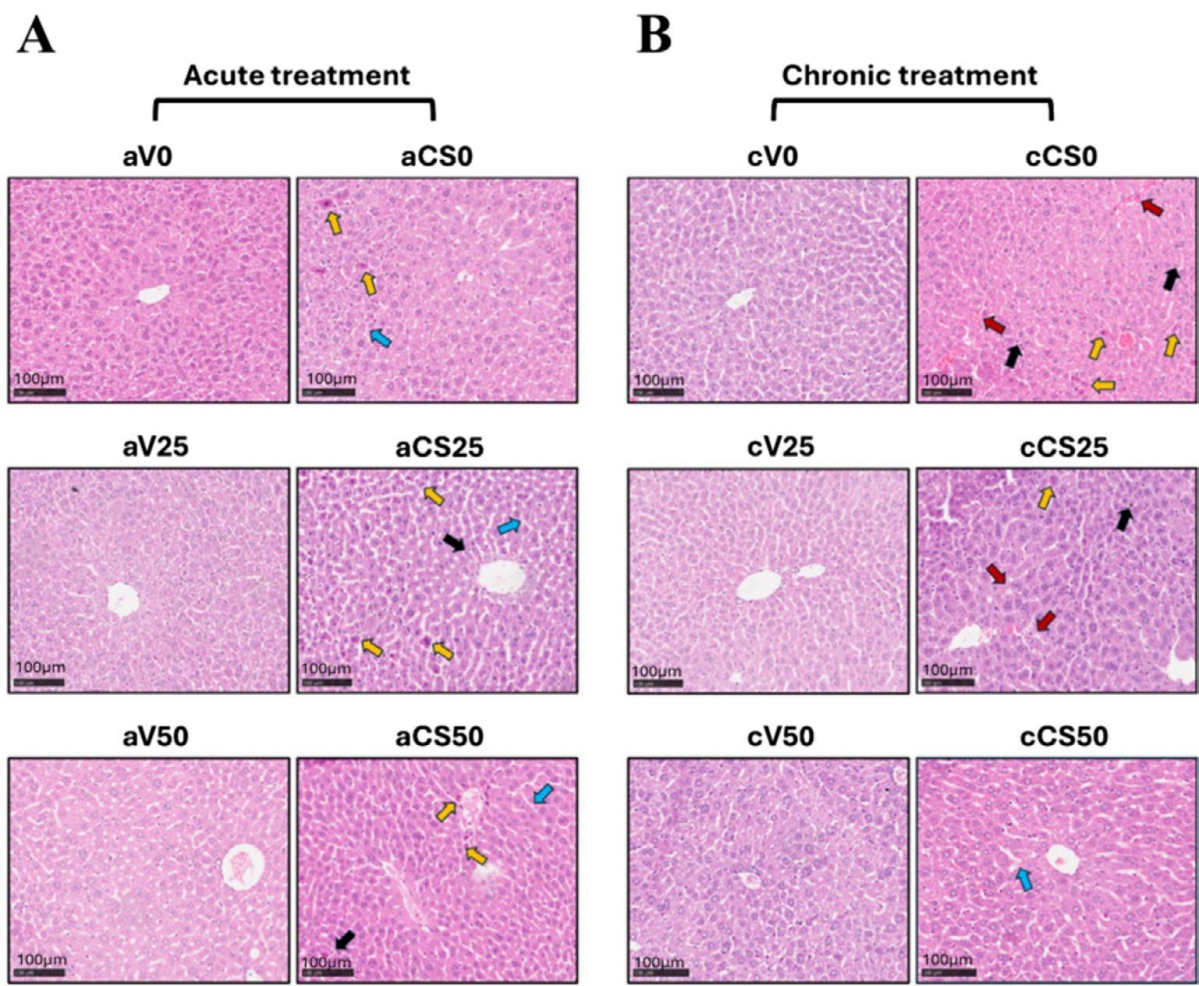
Light microscopy of H&E‐stained liver tissues and quantitative analysis of liver injury scores. (A) H&E staining of liver tissues on acute groups. (B) H&E staining of liver tissues on chronic groups. aV0: Acute ambient air none, aV25: Acute ambient air 25 mg/kg, aV50: Acute ambient air 50 mg/kg, aCS0: Acute cigarette smoke none, aCS25: Acute cigarette smoke 25 mg/kg, aCS50: Acute cigarette smoke 50 mg/kg, cV0: Chronic ambient air none, cV25: Chronic ambient air 25 mg/kg, cV50: Chronic ambient air 50 mg/kg, cCS0: Chronic cigarette smoke none, cCS25: Chronic cigarette smoke 25 mg/kg, cCS50: Chronic cigarette smoke 50 mg/kg. Ambient air groups aV0, aV25, aV50, cV0, cV25, and cV50 exhibited normal histological architecture. Cigarette smoke groups aCS0, aCS25, aCS50, cCS0, and cCS25 displayed mononuclear cell infiltration (black arrows), hemorrhage (red arrows), irregular sinusoidal chords (blue arrows), and eosinophilic necrotic hepatocytes (yellow arrows). cCS50 group showed relatively healthy histological structure. Scale bar = 100 μm.

H&E staining revealed that liver architecture remained normal in ambient‐air groups receiving 0, 25, or 50 mg/kg EGCG, whereas cigarettesmoke groups exhibited significant mononuclear infiltration‐ (black arrow), hemorrhage, irregular sinusoids (blue arrow), and eosinophilic necrosis (yellow arrow), resulting in markedly elevated injury scores (*p* < 0.001) (Figure 1A). Moreover, the sections in cigarette smoke groups chronically treated with EGCG, especially at the dose of 50 mg/kg, displayed relatively healthier liver tissue morphology when compared to cigarette smoke groups acutely treated with EGCG. Notably, chronic EGCG treatment at 50 mg/kg almost completely prevented hemorrhage and necrosis and limited sinusoidal irregularities, yielding significantly lower injury scores than the 0 and 25 mg/kg chronic groups (*p* < 0.001) (Figure 1B).

### 3.2 EGCG Reduces Lipid Peroxidation Markers (MDA)

**FIGURE 2:**
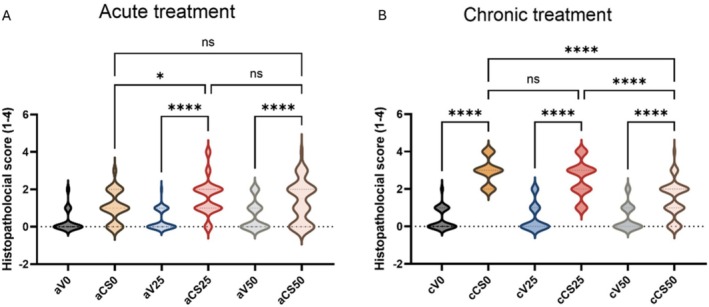
Statistical analysis of liver injury scores across experimental groups. (A) Liver tissues' injury scores on acute groups. (B) Liver tissues' injury scores on chronic groups. Total 100 microscopic areas (*n* = 100) were scored for every experimental group. AV0: Acute ambient air none, AV25: Acute ambient air 25 mg/kg, AV50: Acute ambient air 50 mg/kg, ACS0: Acute cigarette smoke none, ACS25: Acute cigarette smoke 25 mg/kg, ACS50: Acute cigarette smoke 50 mg/kg, CV0: Chronic ambient air none, CV25: Chronic ambient air 25 mg/kg, CV50: Chronic ambient air 50 mg/kg, CCS0: Chronic cigarette smoke none, CCS25: Chronic cigarette smoke 25 mg/kg, CCS50: Chronic cigarette smoke 50 mg/kg. Data are presented the distribution of values, with the median and interquartile range indicated. Statistical significance was determined using one‐way ANOVA followed by Tukey's post hoc test; * = *p* < 0.05, ** = *p* < 0.01, *** = *p* < 0.001, **** = *p* < 0.0001.

**FIGURE 3:**
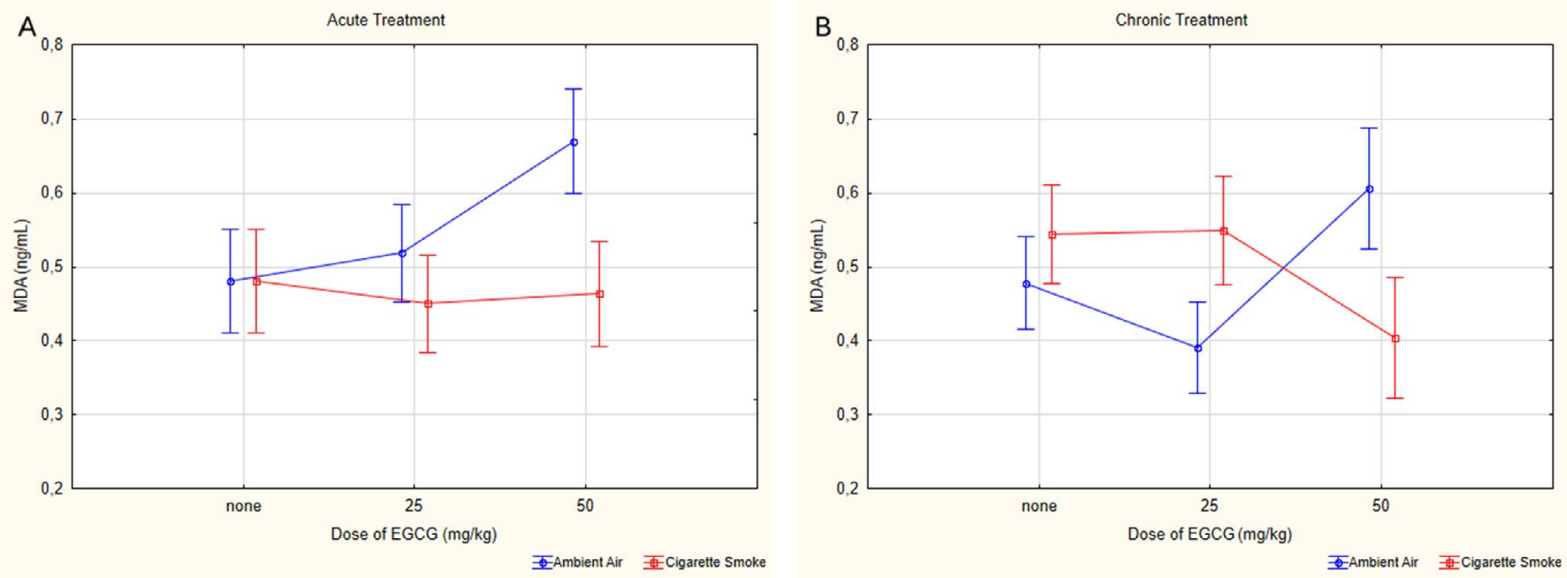
Statistical analysis of MDA levels across experimental groups. (A) MDA levels in acute groups. (B) MDA levels in chronic groups. MDA: Malondialdehyde, EGCG: Epigallocatechin‐3‐gallate, data are presented as mean with 95% confidence interval (CI), aV0: Acute ambient air none, aV25: Acute ambient air 25 mg/kg, aV50: Acute ambient air 50 mg/kg, aCS0: Acute cigarette smoke none, aCS25: Acute cigarette smoke 25 mg/kg, aCS50: Acute cigarette smoke 50 mg/kg, cV0: Chronic ambient air none, cV25: Chronic ambient air 25 mg/kg, cV50: Chronic ambient air 50 mg/kg, cCS0: Chronic cigarette smoke none, cCS25: Chronic cigarette smoke 25 mg/kg, cCS50: Chronic cigarette smoke 50 mg/kg, LSD post hoc test results of groups; aV0 vs. aV50: *p* < 0.001; aV25 vs. aV50: *p* = 0.001; cV0 vs. cV50: *p* = 0.023; cV25 vs. cV50: *p* < 0.001; cCS0 vs. cCS50: *p* = 0.016; cCS25 vs. cCS50: *p* = 0.016; aV50 vs. aCS50: *p* < 0.001; cV25 vs. cCS25: *p* = 0.003; cV50 vs. cCS50: *p* = 0.002; aV25 vs. cV25: *p* = 0.006.

When the effect of group (acute or cronic application), exposure (cigarette smoke or ambient air) and dose (0, 25 or 50 mg/kg application) parameters together (triple interaction) is considered for MDA values, there is no significant effect (*p* = 0,079, *ƞ* ^2^ = 0.075) (Figure 2). The binary interactions of dose‐exposure (*p* < 0.001, *ƞ* ^2^ = 0.305) and group‐exposure (*p* = 0.018, *ƞ* ^2^ = 0.083) showed a significant effect, while there was no significant interaction effect for group‐dose (*p* = 0.209, *ƞ* ^2^ = 0.047). The serum MDA level of the aV50 group was significantly higher than both aV0 (*p* < 0.001) and aV25 (*p* < 0.001) groups. The values of the cV25 group were found to be statistically significantly lower than aV25 (*p* = 0.006) and cV50 (*p* < 0.001), respectively. Based on evaluations made between the groups exposed to cigarette smoke, it was found that the serum MDA level of the cCS25 group was significantly (*p* = 0.016) higher than the cCS50 group (Figure 3A,B).

### 3.3 EGCG Differentially Modulates Levels of Liver Toxicity Markers in Acute Versus Chronic Exposure

**FIGURE 4:**
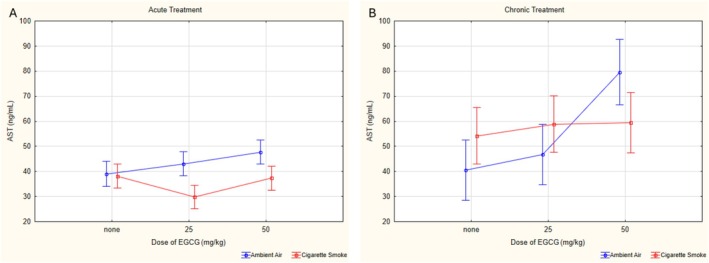
Statistical analysis of AST levels across experimental groups. (A) AST levels on acute groups. (B) AST levels on chronic groups. AST: Aspartate aminotransferase, EGCG: Epigallocatechin‐3‐gallate, data are presented as mean with 95% confidence interval (CI), aV0: Acute ambient air none, aV25: Acute ambient air 25 mg/kg, aV50: Acute ambient air 50 mg/kg, aCS0: Acute cigarette smoke none, aCS25: Acute cigarette smoke 25 mg/kg, aCS50: Acute cigarette smoke 50 mg/kg, cV0: Chronic ambient air none, cV25: Chronic ambient air 25 mg/kg, cV50: Chronic ambient air 50 mg/kg, cCS0: Chronic cigarette smoke none, cCS25: Chronic cigarette smoke 25 mg/kg, cCS50: Chronic cigarette smoke 50 mg/kg, LSD post hoc test results of groups; cV0 vs. cV50: *p* < 0.001; cV25 vs. cV50: *p* < 0.001; aV25 vs. aCS25: *p* = 0.028; cV0 vs. cCS0: *p* = 0.029; cV25 vs. cCS25: *p* < 0.001; cV50 vs. cCS50: *p* = 0.003; aCS0 vs. cCS0: *p* = 0.008; aCS25 vs. cCS25: *p* < 0.001; aV50 vs. cV50: *p* < 0.001; aCS50 vs. cCS50: *p* = 0.001.

When AST values were taken into consideration, group, exposure, and dose showed a significant triple interaction effect (*p* = 0.018, *ƞ* ^2^ = 0.097). The binary interactions of dose‐exposure (*p* = 0.003, *ƞ* ^2^ = 0.139), group‐exposure (*p* = 0.048, *ƞ* ^2^ = 0.049) and group‐dose (*p* = 0.016, *ƞ* ^2^ = 0.101) were found significant. In the ambient air‐exposed groups, the cV50 group had statistically higher AST levels compared to the aV0, cV25, and cV50 groups (*p* < 0.001). Among cigarette smoke‐exposed groups, AST values of the acute groups were significantly lower than those of the chronic groups. Specifically, the aCS0 group had lower levels than the cCS0 group (*p* = 0,008), the aCS25 group was lower than the cCS25 group (*p* < 0,001), and the aCS50 group was lower than the cCS50 group (*p* < 0,001) (Figure 4A,B).

**FIGURE 5:**
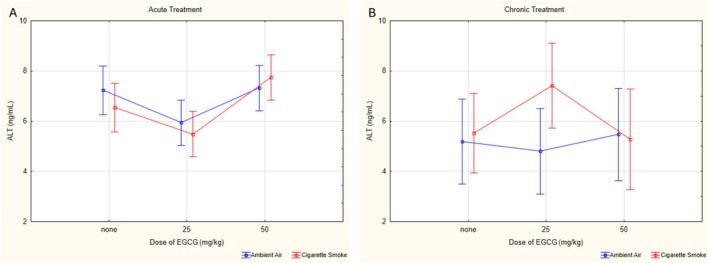
Statistical analysis of ALT levels across experimental groups. (A) ALT levels on acute groups. (B) ALT levels on chronic groups. ALT: Alanine aminotransferase, EGCG: Epigallocatechin‐3‐gallate, data are presented as mean with 95% confidence interval (CI), aV0: Acute ambient air none, aV25: Acute ambient air 25 mg/kg, aV50: Acute ambient air 50 mg/kg, aCS0: Acute cigarette smoke none, aCS25: Acute cigarette smoke 25 mg/kg, aCS50: Acute cigarette smoke 50 mg/kg, cV0: Chronic ambient air none, cV25: Chronic ambient air 25 mg/kg, cV50: Chronic ambient air 50 mg/kg, cCS0: Chronic cigarette smoke none, cCS25: Chronic cigarette smoke 25 mg/kg, cCS50: Chronic cigarette smoke 50 mg/kg, LSD post hoc test results of groups; aV0 vs. aV25: *p* = 0.044; aV25 vs. aV50: *p* = 0.026; aCS25 vs. aCS50: *p* < 0.001; aV0 vs. cV0: *p* = 0.003; aV50 vs. cV50: *p* = 0.006; aCS50 vs. cCS50: *p* = 0.001.

For ALT values, no significant triple interaction (*p* = 0.286, *ƞ* ^2^ = 0.034). Similarly, no significant binary interaction effect was observed for dose‐exposure (*p* = 0.804, *ƞ* ^2^ = 0.006) and group‐ exposure (*p* = 0.275, *ƞ* ^2^ = 0.016) while there was a significant interaction effect for group‐dose (*p* = 0.038, *ƞ* ^2^ = 0.086). The aV0 group had significantly higher ALT levels compared to the aV25 group (*p* = 0.044), while the aV50 group had higher levels than both the aV25 (*p* = 0.026) and cV50 (*p* = 0.006) groups. Among cigarette smoke‐exposed groups, the aCS50 group had significantly higher ALT levels compared to the aCS25 (*p* < 0.001) and cCS50 (*p* < 0.001) groups (Figure 5A,B).

### 3.4 EGCG Enhances Antioxidant Enzyme Activity

**FIGURE 6:**
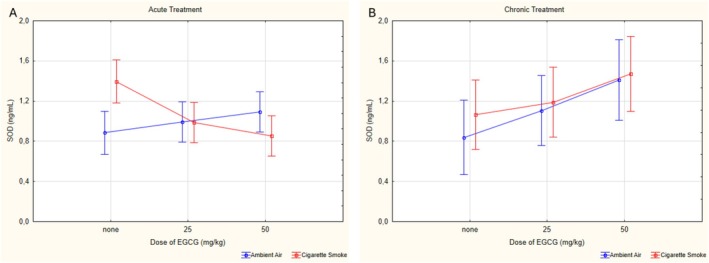
Statistical analysis of SOD levels across experimental groups. (A) SOD levels in acute groups. (B) SOD levels in chronic groups. SOD: Superoxide dismutase, EGCG: Epigallocatechin‐3‐gallate, data are presented as mean with 95% confidence interval (CI), aV0: Acute ambient air none, aV25: Acute ambient air 25 mg/kg, aV50: Acute ambient air 50 mg/kg, aCS0: Acute cigarette smoke none, aCS25: Acute cigarette smoke 25 mg/kg, aCS50: Acute cigarette smoke 50 mg/kg, cV0: Chronic ambient air none, cV25: Chronic ambient air 25 mg/kg, cV50: Chronic ambient air 50 mg/kg, cCS0: Chronic cigarette smoke none, cCS25: Chronic cigarette smoke 25 mg/kg, cCS50: Chronic cigarette smoke 50 mg/kg, LSD post hoc test results of groups; aCS0 vs. aCS25: *p* = 0.048; aCS0 vs. aCS50: *p* = 0.010; cV0 vs. cV50: *p* = 0.011; cCS0 vs. cCS50: *p* = 0.049; aV0 vs. aCS0: *p* = 0.018; aCS50 vs. cCS50: *p* = 0.003.

For SOD values, no significant difference was found in the triple interaction (*p* = 0.373, *ƞ* ^2^ = 0.025). Similarly, no significant binary interaction effect was observed for dose‐exposure (*p* = 0.081, *ƞ* ^2^ = 0.062) and group‐exposure (*p* = 0.843, *ƞ* ^2^ = 0.001) while there was a significant interaction effect for group‐dose (*p* = 0.009, *ƞ* ^2^ = 0.114). In ambient air‐exposed groups, the cV0 group had significantly lower SOD levels than the other groups. Among cigarette smoke‐exposed groups, the aCS0 group had higher SOD levels compared to the aCS25 (*p* = 0.048) and aCS50 (*p* = 0.01) groups. Additionally, the cCS50 group had higher levels than the aCS50 (*p* = 0.003) and cCS0 (*p* = 0.05) groups (Figure 6A,B).

**FIGURE 7:**
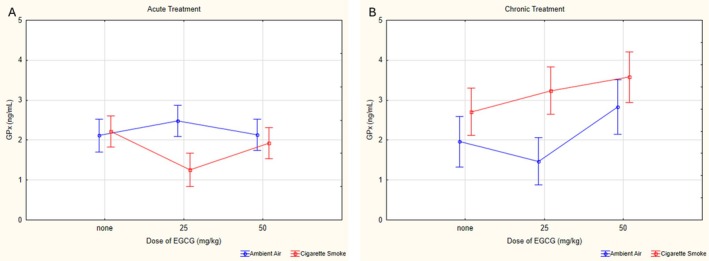
Statistical analysis of GPx levels across experimental groups. (A) GPx levels on acute groups. (B) GPx levels on chronic groups. GPx: Glutathione peroxidase, EGCG: Epigallocatechin‐3‐gallate, data are presented as mean with 95% confidence interval (CI), aV0: Acute ambient air none, aV25: Acute ambient air 25 mg/kg, aV50: Acute ambient air 50 mg/kg, aCS0: Acute cigarette smoke none, aCS25: Acute cigarette smoke 25 mg/kg, aCS50: Acute cigarette smoke 50 mg/kg, cV0: Chronic ambient air none, cV25: Chronic ambient air 25 mg/kg, cV50: Chronic ambient air 50 mg/kg, cCS0: Chronic cigarette smoke none, cCS25: Chronic cigarette smoke 25 mg/kg, cCS50: Chronic cigarette smoke 50 mg/kg, LSD post hoc test results of groups; aCS0 vs. aCS25: *p = 0.009; cV0* vs. *cV50: p* = 0.027; cV25 vs. cV50: *p* = 0.001; cCS0 vs. cCS50: *p* = 0.018; aV25 vs. aCS25: *p* = 0.001; cV0 vs. cCS0: *p* = 0.042; cV25 vs. cCS25: *p* < 0.001; aV25 vs. cV25: *p* = 0.005; aCS25 vs. cCS25: *p* < 0.001; aCS50 vs. cCS50: *p* < 0.001.

For GPx values, a significant difference was found in the triple interaction (*p* = 0.003, *ƞ* ^2^ = 0.141). Similarly, a significant binary interaction effect was observed for group‐exposure (*p* < 0.001, *ƞ* ^2^ = 0.258) and group‐dose (*p* = 0.023, *ƞ* ^2^ = 0.092) while there was no significant interaction effect for dose‐exposure (*p* = 0.882, *ƞ* ^2^ = 0.003). In ambient air‐exposed groups, the cV25 group had significantly lower GPx levels compared to the cV50 (*p* < 0.001) and aV25 (*p* = 0.005) groups, whereas the cV50 group had higher levels than the cV0 group (*p* = 0.027). Among cigarette smoke‐exposed groups, the aCS25 group had significantly lower GPx levels compared to the cCS25 (*p* < 0.001) and aCS0 (*p* = 0.009) groups. The cCS50 group exhibited higher levels compared to the cCS0 (*p* = 0.018) and aCS0 (*p* < 0.001) groups (Figure 7A,B).

**FIGURE 8:**
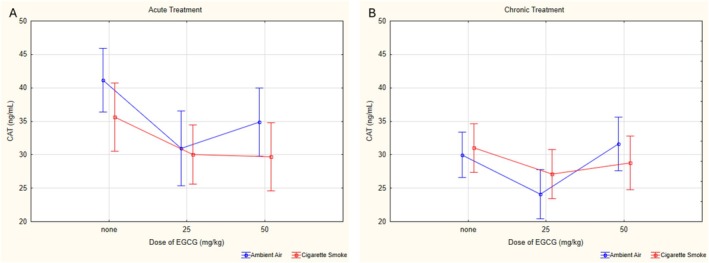
Statistical analysis of CAT levels across experimental groups. (A) CAT levels in acute groups. (B) CAT levels in chronic groups. CAT: Catalase, EGCG: Epigallocatechin‐3‐gallate, data are presented as mean with 95% confidence interval (CI), aV0: Acute ambient air none, aV25: Acute ambient air 25 mg/kg, aV50: Acute ambient air 50 mg/kg, aCS0: Acute cigarette smoke none, aCS25: Acute cigarette smoke 25 mg/kg, aCS50: Acute cigarette smoke 50 mg/kg, cV0: Chronic ambient air none, cV25: Chronic ambient air 25 mg/kg, cV50: Chronic ambient air 50 mg/kg, cCS0: Chronic cigarette smoke none, cCS25: Chronic cigarette smoke 25 mg/kg, cCS50: Chronic cigarette smoke 50 mg/kg, LSD post hoc test results of groups; aV0 vs. aV25: *p* = 0.002; aV0 vs. aV50: *p* < 0.001; cV25 vs. cV50: *p* = 0.024; aV0 vs. cV0: *p* < 0.001; aV25 vs. cV25: *p* = 0.040.

For CAT values, no significant difference was found in the triple interaction (*p* = 0.787, *ƞ* ^2^ = 0.008). Similarly, no significant binary interaction effects were observed for dose‐exposure (*p* = 0.266, *ƞ* ^2^ = 0.042), group‐ exposure (*p* = 0.100, *ƞ* ^2^ = 0.044) and group‐dose (*p* = 0.182, *ƞ* ^2^ = 0.054). Only, significant differences were detected for the main effects of group (*p* < 0.001, *ƞ* ^2^ = 0.200) and dose (*p* < 0.001, *ƞ* ^2^ = 0.225) except for exposure (*p* = 0.173, *ƞ* ^2^ = 0.030). The aV0 group had significantly higher CAT levels than the aV25 (*p* = 0.002), aV50 (*p* = 0.04), and cV0 (*p* = 0.001) groups. The cV50 group had significantly higher CAT levels compared to the cV25 group (*p* = 0.024). In cigarette smoke‐exposed groups, the aCS25 group had significantly lower CAT levels compared to the cCS25 group (*p* = 0.004) (Figure 8A,B).

## 4 Discussion

Oxidative stress is defined as the disruption of the balance between the production of free radicals by cells and the prevention of damage by these molecules. The liver is involved in the oxidation of carbohydrates, fats, and products. This makes it sensitive to oxidative stress damage. ROS is one of the main causes of liver damage (Pagliara et al. 2003).

Catechins are the best‐known ROS scavengers (Robak and Gryglewski 1988) and EGCG, as the most abundant catechin in green tea, is an effective free radical scavenger thanks to its dihydroxyl and trihydroxyl structures (Rice‐Evans 1999). It has a protective effect against lipid peroxidation in oxidative stress situations and can increase antioxidant levels in the cell and can play a role in the elimination of free radicals. Lakshmi et al. (2020) found that exposure to cigarette smoke increased oxidative stress by elevating ROS levels in airway epithelial cells, and EGCG attenuated this effect. Post‐EGCG administration dose‐dependently decreased ROS production and accumulation. It also suppressed membrane lipid peroxidation. However, the questions of whether smoke exposure indirectly affects the liver and whether EGCG can confer similar protection in vivo remain to be addressed. It is known that cigarette smoke has cell damage and genotoxic effects in the liver (Bandyopadhyaya et al. 2008). Therefore, we hypothesized that EGCG may be effective in protecting the liver from the oxidative effects of cigarette smoke. The main results of the present study are: (I) The administration of EGCG during prolonged exposure to cigarette smoke does not exhibit an effect on the inflammatory markers associated with liver pathology, (II) Besides, the sections from cigarette smoke groups chronically treated with EGCG, especially at the dose of 50 mg/kg, displayed relatively healthier liver tissue morphology when compared to cigarette smoke groups acutely treated with EGCG. This suggests that chronic treatment withf EGCG has a protective effect on cigarette smoke‐induced liver damage, (III) Chronic administration of EGCG effectively reduced oxidative stress caused by prolonged exposure to harmful components of cigarette smoke, thereby providing a potential protective effect. This application also significantly increased the levels of antioxidant enzymes such as glutathione peroxidase (GPx) and superoxide dismutase (SOD), both critical in the cellular defense against oxidative damage, (V) It is essential to acknowledge that the beneficial effects of EGCG administration on the antioxidant defense system require an adjustment of dose and duration of time to manifest fully; it is noteworthy that acute applications of this compound have yielded no successful outcomes in the short term.

Mechanistically, EGCG elevates both extracellular and intracellular reactive oxygen species (ROS), but the kinetics and consequences of these increases differ markedly. Whereas extracellular ROS generated by EGCG peak early and then wane, Tao et al. (2014) found that intracellular ROS in oral cancer cells continued to rise even after EGCG was no longer detectable in the cytosol or culture medium by 12 h‐indicating a sustained intracellular oxidative response. For this effect, EGCG requires the mitochondrial electron transport system and can act as a prooxidant to generate ROS that can oxidize the cysteine thiol of Keap1, releasing Nfr2 (Na and Surh 2006). In the presence of abundant antioxidant enzymes, this stimulation can be blocked because ROS can be eradicated from the environment by them. This mechanism may help to explain the reduced antioxidant levels observed in the acute treatment groups. The rapid elimination of ROS by existing antioxidant enzymes may have limited the sustained activation of endogenous antioxidant pathways. In our study, antioxidant enzyme levels in acute groups were found to be similar to or lower than those in the control groups. In the acute groups where cigarette smoke and EGCG were administered together, SOD and GPx enzyme levels decreased. The oxidants present in EGCG and cigarette smoke exerted pressure on the antioxidant system, leading to increased oxidative stress. Consequently, the levels of antioxidant enzymes were reduced to balance redox homeostasis again. Wu et al. (2006) investigated the antioxidant mechanisms of EGCG on endothelial cells and demonstrated that EGCG reduces oxidative stress by upregulating heme oxygenase‐1 via the PI3K/Akt and ERK signaling pathways. In their time‐course experiments, they found that the expression of key antioxidant enzymes began to rise at six h post‐treatment and reached maximal levels by 12 h. Another study by Chen et al. (2000) extended these findings in hepatoma cells, demonstrating that EGCG activates MAPK signaling peaked in 2 h, which in turn drives AREmediated gene expression of Phase II detoxifying enzymes. Although they noted that EGCG can activate the Nrf2 pathway, they did not assess the link that Nrf2 effects on enzymes such as SOD and GPx, nor did they examine any potential toxicity. In the clinical study, it was found that EGCG reached the highest level in human blood 2 h after oral ingestion (Unno et al. 1996). The data of the acute application groups were obtained after 180 min exposure to cigarette smoke in our study. However, since there was enough time for the increased transcription of antioxidant enzymes, time is not enough for increased expression of antioxidant level to observe in the acute groups. Another results which we obtained in the acute groups is that the oxidative stress in the groups exposed to ambient air and 50 mg/kg EGCG administration observed significant increase. In parallel, James et al. (2015) reported that acute high‐dose EGCG also decreased mRNA expression of GR, supporting their observation of an initial suppression of the antioxidant defense system under acute high‐dose EGCG treatment (James et al. 2015). In sharp contrast, the mitochondrial prooxidant effect triggered by acute, high‐dose EGCG inflicts irreversible damage on the host cell, may cause the cell death.

EGCG itself increased oxidative stress within the cell, either by increasing ROS production or by acting as an electrophile itself (Xu et al. 2005). When oxidative stress induced by EGCG was sufficient to activate the Nrf2 pathway (Wu et al. 2006), an increase in antioxidant enzyme transcription was observed. This response is known to be triggered by oxidative or electrophilic stress (Wu et al. 2006; Itoh et al. 2004). EGCG appears to target mitochondria to trigger a selfamplifying ROS cascade that ultimately induces mitochondrial antioxidant defenses. Intracellular ROS first appear in mitochondria within 1 h of EGCG treatment—consistent with the “ROSinduced ROS release” (RIRR) model. At the initial phase of this model, mitochondrial perturbation opens mPTP channels and releases ROS into the cytosol, propagating further ROS generation in neighboring mitochondria (Tao et al. 2014; Brady et al. 2006; Zorov et al. 2000, 2006). By contrast, extracellular ROS levels rise rapidly during the initial hours of exposure and subsequently decline. This transient extracellular burst serves to prime neighboring cells. The released ROS act as signaling molecules that stimulate antioxidant enzyme production in surrounding tissue, enabling rapid clearance of oxidants from the environment (Zorov et al. 2000). All these studies' findings underscore EGCG's capacity to orchestrate a robust Nrf2driven antioxidant and detoxification program in smoke‐stressed liver tissue. EGCG induces AREmediated gene expression in mouse liver, confirming its activation of Nrf2 signaling‐ (Chen et al. 2000; Shen et al. 2005). Under basal conditions, Nrf2 remains inactive in the cytosol through its association with Keap1, which prevents its nuclear migration (Itoh et al. 1999). Upon oxidative stress or AREinducing stimulation, Nrf2 is released from Keap1 and translocates into the nucleus to drive gene expression (Dinkova‐Kostova et al. 2002). Phase II detoxifying enzymes or antioxidants have a sequence with an enhancer region called ARE. It stimulates the gene expression of Phase II detoxifying enzymes and increase in antioxidant levels (Xie et al. 1995). Activation of Phase II detoxifying enzymes and antioxidant genes is mediated by the binding of Nrf2 to ARE, which are present in many detoxification genes—including glutathione S‐transferase (GST) family members (Lee and Surh 2005). Another study showed that Nrf2 is a key transcription factor regulating gene expression under the control of the ARE region. EGCG was also found to transcriptionally regulate the ARE‐reporter gene (Chen et al. 2000), Thus, antioxidant and phase II enzyme activities of cells are also increased (Rubiolo et al. 2008) (Chen et al. 2000).

Although acute EGCG treatment elevated oxidative marker and failed to enhance antioxidant enzymes, prior studies highlight adaptive responses under longer regimens. Kim et al. (2000) observed that plasma EGCG initially rises for four days of dietary greentea intake before declining to baseline over the subsequent ten days, while Shen et al. (2005) demonstrated in vivo induction of Phase II and antioxidant‐response genes following chronic exposure. Such adaptive metabolic shifts may underlie EGCG's self‐modulation of bioavailability, as chronic EGCG or green tea intake can influence the Phase II biotransformation and subsequent exposure of this compound (Lambert et al. 2010). James et al. (2015) found that twoweek dietary pretreatment (3.2 mg/g diet) reduced plasma EGCG exposure by 57% and hepatic exposure by 71%, thereby preventing the ALTelevating hepatotoxicity induced by a 750 mg/kg acute oral dose. Notably, EGCG‐induced hepatotoxicity has been associated with oxidative stress, including a fivefold increase in hepatic lipid peroxidation (Lambert et al. 2010). In our study, as EGCG was administered via intraperitoneal injection—a route known to yield higher bioavailability—at just 50 mg/kg once daily for 21 days, achieving comparable antioxidant and anti‐inflammatory effects compared to acute treatment, underscoring its dose and durationdependent protective effects in liver tissue. When evaluated together with these data, it is seen that although EGCG administration in the acute period might be sufficient to induce oxidative stress to stimulate the Nrf2‐ARE pathway, it's possibly not sufficient for the expression of the antioxidant defense system to appear. The mitochondrial ROS surge can activate redoxsensitive transcription factors such as Nrf2, leading to the upregulation of mitochondrial antioxidant enzymes (e.g., SOD, GPx) observed in our chronic EGCG regimen. In order to observe the effects of EGCG application on the levels of antioxidant enzymes depending on time, we have formed 21‐day chronic application groups. The data obtained from these groups show that the positive effects of EGCG occur depending on time and dose. In studies conducted in cell culture, when cells were exposed to EGCG alone, low doses stimulated ARE activation, while high doses appeared to inhibit it. In two studies, the effect of EGCG varied depending on the doses (Chen et al. 2000; Kweon et al. 2006). In the current study, chronic administration of 50 mg/kg dose of EGCG significantly increased antioxidant SOD and GPx enzyme levels. Chronic administration of 25 mg/kg EGCG dose had no positive effect on SOD and GPx levels. Chronically administration of 50 mg/kg dose was determined to be necessary to achieve the desired effect on the antioxidant system. The increase antioxidants positively impacted liver toxicity scores in the group exposed to cigarette smoke and chronically administered with a 50 mg/kg dose of EGCG. We observed significantly less liver injury in cigarette smoke‐exposed groups that underwent chronic EGCG treatment, particularly at a dose of 50 mg/kg, compared to those that received acute EGCG treatment. EGCG can have antioxidant (Rice‐Evans 1999) as well as prooxidant properties (Sergediene et al. 1999). Notably, 25 mg/kg is an insufficient dose for the desired response, whereas 50 mg/kg EGCG dose demonstrated efficacy in mitigating oxidative stress markers, underscoring its dose‐dependent bioactivity. Our results indicate that EGCG may exhibit a a prooxidant effect, increasing antioxidant enzyme levels, and a longer time is required for the increase in enzyme levels compared to acute administration.

These findings align with the hypothesis that chronic EGCG treatment is essential to harness its antioxidant potential, as acute interventions fail to sustain enzymatic upregulation. This dual nature of EGCG's effects suggests that its efficacy depends on both dosage and the body's oxidative stress load—a phenomenon consistent with prior reports on polyphenolic compounds. In support of this, Al‐Awaida et al. (2023) observed in their 90‐day study that chronic EGCG administration at comparable doses led to a significant decline in inflammatory markers and antioxidant gene expression, corroborating our conclusions regarding the necessity of prolonged treatment for optimal therapeutic outcomes. Importantly, their findings reinforce the validity of our data, particularly the superiority of the 50 mg/kg dose and the critical role of chronic administration in achieving antioxidative and anti‐inflammatory benefits.

EGCG administration failed to enhance CAT activity in a dose‐ or time‐dependent manner. In a mouse study, GPx enzyme levels increased in the liver, whereas CAT levels remained unchanged (Tanii et al. 2008). CAT, SOD1, and GPx are present in the cytosol (Pervaiz et al. 2009), however, the majority of GPx and the Mn‐dependent SOD2 isoform—localize to mitochondria, whereas Cu/Zn‐SOD1 remains cytosolic (Lawler and Powers 1998). Given that EGCG requires the mitochondrial electron transport system, and the amount of ROS produced by it (Wu et al. 2006). Under the light of this information, its selective upregulation of mitochondrial GPx and SOD activities, but not cytosolic CAT, is consistent with a primary mitochondrial site of action.

ROS–mediated lipid peroxidation generates a variety of reactive aldehydes, such as 4‐HNE, MDA, propanal, and hexanal (Ayala et al. 2014). Among these, MDA is a principal end product, and its levels rise markedly under heavy‐metal stress. For example, Patra et al. (2001) reported significant increases in hepatic and cerebral MDA after four weeks of subchronic lead exposure in rats, while Pagliara et al. (2003) showed that lead‐induced oxidative stress led to Kupffer cell apoptosis and hepatic hyperplasia. In a separate investigation, rat livers exposed to lead similarly exhibited elevated MDA levels in parallel with enhanced lipid peroxidation (Annabi Berrahal et al. 2007). In our study, acute or chronic administration of 50 mg/kg EGCG increased MDA levels in the absence of cigarette smoke. However, when the same dose was administered chronically during cigarette smoke exposure, MDA levels decreased. Histopathological examination revealed that EGCG did not increase the histopathological score in mice exposed to ambient air. We propose that the beneficial effects of chronic EGCG are dependent on preexisting oxidative stress. As demonstrated by Srividhya et al., administration of 2 mg/kg EGCG (i.g.) for 30 days decreased hepatic lipid peroxides and protein carbonyls and increased both small‐molecule antioxidants and antioxidant enzymes only in aged rats with preexisting oxidative stress, not in 3‐ 4‐month‐old young animals (Srividhya et al. 2008). These findings can explain why administering 50 mg/kg EGCG failed to enhance antioxidant status in the absence of preexisting oxidative stress. In our study, chronic cigarette smoke exposure provided the necessary preexisting oxidative stress, thereby allowing the 50 mg/kg EGCG regimen to demonstrate significant hepatic improvement under this condition.

James et al. reported that high‐dose oral administration of EGCG led to an 80‐fold increase in plasma alanine aminotransferase (ALT), a 59% reduction in reduced hepatic glutathione, a 33% reduction in total hepatic glutathione, and elevated levels of phosphorylated histone H2AX in liver tissue. Compared with the 750 mg/kg dose used by James et al., which elicited hepatotoxicity, our substantially lower dosing regimens were not expected to cause liver injury; accordingly, we observed no ALT elevation at any dose. An elevated AST was noted only in the group receiving chronic ambient air plus 50 mg/kg EGCG. AST is also found in the heart (Pratt and Kaplan 2000), skeletal muscle, kidney, brain, pancreas, spleen, lung, and erythrocytes (Pratt and Kaplan 2000; Ndrepepa 2021). ALT is a more specific parameter in evaluating liver damage. Therefore, MDA and AST are not as liver‐specific as ALT (Wen et al. 2013). Inflammatory data are supported by histopathological examinations. Histopathological examination revealed that the application of two different doses of EGCG in the ambient air groups did not alter the histopathological scores, yielding results similar to the control group that did not receive EGCG. These findings indicate that EGCG administration under our conditions does not exhibit toxic effects on the liver. However, further studies are needed to elucidate why AST levels increased and what effects EGCG has on other organs.

‐Taken together, EGCG's early prooxidant action within targeted cells paradoxically promotes an indirect antioxidant defense in adjacent cells. Whether the net outcome is protective or deleterious depends critically on both the administered dose and the duration of exposure. Together, these data clarify how EGCG both elicits and then counterbalances oxidative signals to enhance mitochondrial resilience in smoke‐stressed liver tissue.

This study has certain limitations. This study represents our initial effort to examine whether EGCG can prevent the harmful effects of cigarette smoke on the liver and how these effects vary with dose and administration schedule. Due to the scope of our study, we prioritized these core aims when designing the experiments. In future work, it will be investigated how specific signaling pathways are affected by chronic EGCG treatment and explored how mitochondrial and cytosolic antioxidant dynamics are altered using additional techniques, such as molecular techniques, functional and behavioral readouts. Future studies should explore how cigarette smoke increases oxidative stress and induces cell death. Additionally, the effects of EGCG on these pathways, particularly through the Nrf2 signaling pathway, should be examined in detail to clarify how chronic EGCG administration counteracts the harmful effects of cigarette smoke. Another limitation is that our evaluations were solely based on liver tissue. Although chronic EGCG under ambient‐air conditions did not alter liver histology or serum markers, the absence of detailed mechanistic assays in normal (non‐ambient air or cigarette smoke administered) tissues limits our understanding of its long‐term safety profile. Future studies will include extended follow‐up in both hepatic and extra‐hepatic organs to assess the potential side effects of prolonged EGCG administration in the absence of oxidative challenges (Lakshmi et al. 2020; Chen et al. 2000; Wu et al. 2006; Kweon et al. 2006). It is necessary to reveal and reinforce the effect and potential of EGCG by increasing the number of studies that use additional animal models to evaluate the effectiveness of EGCG, conduct clinical trials, assess the dose and duration of administration, and evaluate factors that increase external oxidative stress together. After these studies, we think that the administration of EGCG at the right dose specific to the individual will have important results for pre‐/post‐clinical treatment. The chronic effects of EGCG on other tissues should also be investigated. Furthermore, only the effects of 25 and 50 mg/kg doses i.p. were analyzed in this study. The potential effectiveness of other doses without causing any harm, especially in acute administration, should be explored. Given that antioxidant enzyme levels increased in the mitochondria while cytosolic enzymes like CAT remained unchanged, EGCG is thought to exert its effects primarily through mitochondrial mechanisms. Additionally, cigarette smoke may enhance red blood cell production and degradation, which leads to iron accumulation and potentially contributes to ferroptosis‐related organ damage. Lastly, biochemical parameters in this study were obtained from serum samples. To determine whether EGCG has any toxic effects, future studies should incorporate direct tissue measurements to identify which organs may be affected.

## 5 Conclusion

‐Chronic EGCG administration at 50 mg/kg significantly reduced cigarette smoke‐induced oxidative stress and induced mitochondrial antioxidant enzymes—likely via the Nrf2–GPx4 axis, thereby reducing lipid peroxidation and preserving redox homeostasis under cigarette smoke exposure, whereas acute EGCG treatment failed to enhance antioxidant activity and even transiently elevated oxidative markers. These results underscore EGCG's promise as a protective agent against smoke‐induced hepatic oxidative damage. However, several limitations should be noted: we did not directly assay Nrf2 activation or ferroptosis biomarkers, our analyses were restricted to liver tissue and serum without EGCG‐only control groups, and only two dose levels were tested. The long‐term effects of EGCG in the absence of external oxidants—and its impact on nonhepatic tissues—remain to be elucidated.

## Author Contributions

**Kağan Ağan:** conceptualization (equal), data curation (equal), funding acquisition (equal), investigation (lead), methodology (equal), project administration (lead), writing – original draft (lead), writing – review and editing (lead). **Şerif Demir:** funding acquisition (equal), supervision (lead), writing – review and editing (equal). **Recep Özmerdivenli:** conceptualization (equal). **Aydan Fülden Ağan:** investigation (equal), project administration (supporting), visualization (lead), writing – review and editing (supporting). **Ali Tuğrul Akın:** formal analysis (equal), investigation (equal). **Merve Alpay:** conceptualization (equal), methodology (equal), writing – review and editing (equal). **Özge Beyazçiçek:** investigation (supporting). **Ersin Beyazçiçek:** investigation (supporting). **Mehmet Ali Sungur:** formal analysis (lead).

## Ethics Statement

The Animal Research Ethics Committee of Duzce University approved the present study (protocol number: 2019/3/1). Experiments were performed in accordance with the Guide for the Care and Use of Laboratory Animals, published by the US National Institutes of Health (NIH publication No. 85–23, revised 1996).

## Conflicts of Interest

The authors declare no conflicts of interest.

## Data Availability

Data will be made available on request.
